# Co-Dispersion Behavior of ZrB_2_–SiC–B_4_C–C Powders with Polyethyleneimine

**DOI:** 10.3390/ma6094249

**Published:** 2013-09-23

**Authors:** Jie Yin, Jian Chen, Xuejian Liu, Hui Zhang, Yongjie Yan, Zhengren Huang, Dongliang Jiang

**Affiliations:** 1 State Key Laboratory of High Performance Ceramics and Superfine Microstructure, Shanghai Institute of Ceramics, Chinese Academy of Sciences, Shanghai 200050, China; E-Mails: nannancj@mail.sic.ac.cn (J.C.); xjliu@mail.sic.ac.cn (X.L.); zhanghui@mail.sic.ac.cn (H.Z.); dljiang@mail.sic.ac.cn (D.J.); 2 Engineering Ceramics Department, Korea Institute of Materials Science (KIMS), 797 Changwondaero, Changwon, Gyeongnam 642-831, Korea

**Keywords:** ZrB_2_, dispersion, suspension, surface, rheological behavior

## Abstract

The aqueous dispersion behavior of ZrB_2_, SiC powders with B_4_C and C as sintering aids was investigated. Well co-dispersed suspension can be obtained in acidic solutions in presence of polyethyleneimine (PEI). The adsorption of PEI on the powder surface was measured by thermal gravimetric (TG) analysis. Rheological measurements displayed the effect of dispersant on the flow behavior of as-prepared slurries. An optimum condition was obtained with 1 wt % PEI. The viscosity of 40 vol % ZrB_2_–SiC–B_4_C–C (ZSBC) suspension at 100 s^−1^ was as low as 0.74 Pa·s, which was suitable for aqueous processing.

## 1. Introduction

Colloidal processing is an attractive fabricating method to prepare near-net-shape, and cost-effective ceramic components with homogeneous structures [1,2]. Colloidal processing has been applied to prepare oxide ceramics (Al_2_O_3_ [2]), carbide ceramics (SiC [3]), and nitride ceramics (Si_3_N_4_ [4]). Being the promising candidates for potential use in various applications, *i.e.*, thermal protection systems for hypersonic aerospace vehicles, high-temperature electrodes, and crucibles for molten metal contact [5,6,7], ZrB_2_-based ceramics have been attracting increasing attentions in recent years. Investigations on the wet-forming of ZrB_2_-based ceramics have been conducted, yet the properties of as-fabricated ceramics were inferior to those prepared by conventional consolidation methods [8]. In colloidal processing, the dispersion behavior of ceramic powders proves to be the limiting factor, affecting the fluidity as well as the homogeneity of suspensions, and therefore the mechanical properties of sintered bodies [9].

It is believed that the addition of SiC could improve the oxidation resistance and mechanical properties compared with monolithic ZrB_2_ ceramics [10,11]. Moreover, since the presence of an oxide layer on the powder surface inhibits its high-temperature densification progress, sintering aids such as B_4_C [12] and carbon [13] are usually required. On the other hand, previous researchers used polyethyleneimine (PEI) to disperse monolithic ZrB_2_ effectively [14]. PEI was reported in several studies to be an appropriate dispersant for SiC [15] and B_4_C [16] powders. However until now, the aqueous co-dispersion behavior of a ZrB_2_–SiC–B_4_C–C (ZSBC) powder system had rarely been reported [17], which is of crucial importance to the subsequent consolidation process.

In the present work, the properties of ZSBC suspension are investigated in terms of surface charge, adsorption, and rheological behavior, in order to obtain a well co-dispersed suspension.

## 2. Experimental Procedure

Commercially available ZrB_2_ powder (Dandong Chemical Co. Ltd., Dandong, China, the average particle size was 2.5 μm, multilateral shape, and the specific area was 5.24 m^2^·g^−1^), α-SiC powder (FCP 15C, SIKA TECH., Lillesand, Norway, multilateral shape, with a purity of >99% and an average particle size of <0.5 μm), B_4_C powder (Mudanjiang Jingangzuan Boron Carbide Co. Ltd., Mudanjiang, China, multilateral shape, and the average particle size was <1 μm), and water-soluble carbon black (Shanghai Yingchang Chemical Co. Ltd., Shanghai, China, spherical shape, and the average particle size was <1 μm) were used as raw materials. PEI (30 wt % aqueous solution, TCI, P0381) was selected as dispersant.

ZrB_2_ powder was attrition milled (Model 01-HD, Union Process Precision Machinery Co. Ltd., Qingdao, China) at 300 rpm for 2 h in ethanol medium, in a Teflon-coated tank, using cobalt-bonded WC media and a cobalt-bonded WC spindle. The particle size was refined to 0.32 μm after milling. In order to remove surface oxide impurity, ZrB_2_ was further washed with a hydrochloric acid solution (1:1 in weight ratio) and freezing dried (FD-1A-50, Beijing Boyikang Experimental Instrument Co. Ltd., Beijing, China). The aqueous slurries were prepared as follows: ZrB_2_, SiC, B_4_C, and C powders, together with PEI (all contents were based on the weight percentages of dry powders), were dispersed into water, and then stirred at 400 rpm for 1 h.

Zeta potential was measured with Zetaplus (Brookhaven Instruments Corp., Holtsville, NY, USA), and suspensions of 0.01 vol % solids content were prepared with and without dispersants. The composition ratio of the ZSB(C) powders was as follows: ZrB_2_–20 vol % SiC–2 wt % B_4_C(–1 wt % C). The adsorption of PEI on the ZSBC particle surfaces was tested by thermogravimetry analysis (TG, Netzsch STA 449C, Selb, Germany) in a nitrogen atmosphere. Before the measurements, 40 wt % ZSBC suspensions containing 2 wt % PEI were ball milled for 48 h, then the particles were separated from the supernatants by centrifuging at 10,000 rpm for 60 min, and freeze dried afterwards. As for strong acid-etching treatment (for surface adsorption measurement), ZSBC powders were firstly dispersed into HNO_3_ solution at a mass ratio of 1:10, stirred for 48 h, and then filtrated and freeze dried.

The shear-dependent behavior of the ZrB_2_-based suspension systems was evaluated by ascending and descending shear rate, ramped from 0 to 300 s^−1^ and from 300 s^−1^ to 0, respectively (with 300 s^−1^ maintaining for 50 s).

## 3. Results and Discussion

### 3.1. Electrophoretic Property of ZSBC Suspensions

**Figure 1:**
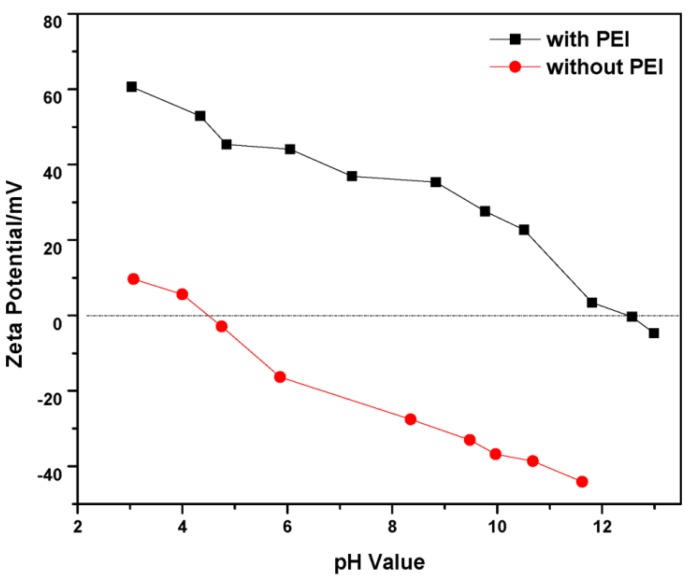
Zeta potential of ZrB_2_ suspensions with and without presence of polyethyleneimine (PEI).

Figure 1 shows the zeta potential of ZrB_2_ slurries. The isoelectric point (IEP) of ZrB_2_ was ~4.5, which was close to the previous reported values of ~4.7 [18]. The surface charge of ZrB_2_ was negative when pH was higher than the IEP. However the IEP of ZrB_2_ shifted towards a basic region in the presence of PEI. This is due to the fact that PEI ionizes in the acid condition, carries a positive charge, and adsorbs onto the particle surface via electrostatic force (hydrogen bonding is proposed to be the dominant mechanism). The dispersion of ZrB_2_ in the water could be attributed to the surface oxide layer, which was illustrated by earlier researchers [18].

**Figure 2:**
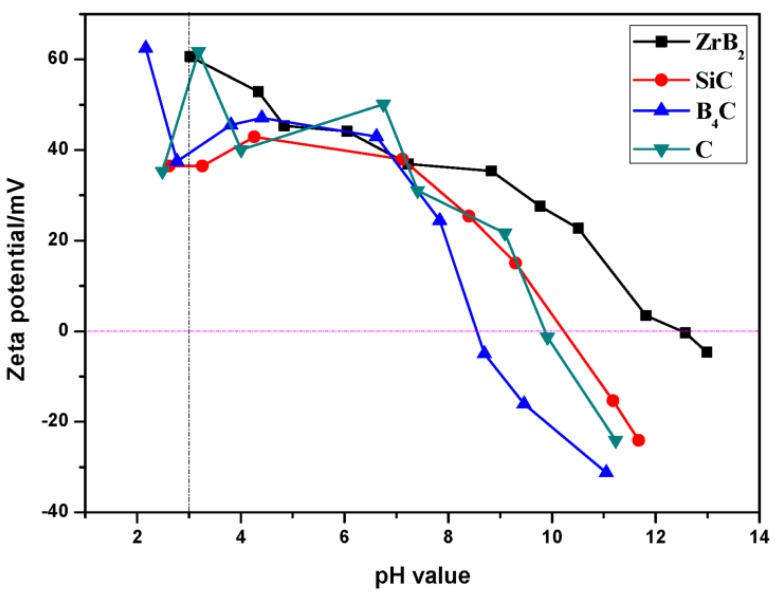
Zeta potential of ZrB_2_, SiC, B_4_C, and C suspensions in the presence of PEI.

In the current work, not only ZrB_2_, but also other powders were concerned, since the co-dispersion behavior of ZrB_2_, SiC, B_4_C, and C is critical to obtain green bodies with chemical homogeneity. Figure 2 displays the zeta potentials of ZrB_2_, SiC, B_4_C, and C powders in the presence of PEI. The maximum absolute values of zeta potential appeared in the acidic condition (all >30 mV at pH ≈ 3), which manifested the best co-dispersion status. It is worth noting that the surface chemistry and dispersion behavior of PEI onto SiC powder has already been reported by previous researchers, and that they believed that an acid solution was the best dispersing medium [15]. Thus the ZSBC powders can be well co-dispersed in an acidic solution, in the presence of PEI.

### 3.2. Adsorption Studies

**Figure 3:**
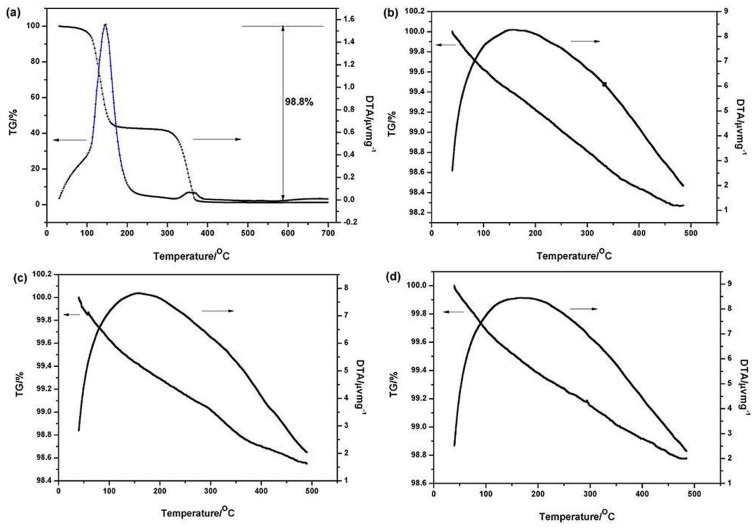
Thermal behavior of (**a**) PEI and (**b–d**) ZSBC-PEI at different pH values: (**b**) pH 3.2; (**c**) pH 6.6; (**d**) pH 10.6.

The adsorption of PEI onto the particle surfaces would evidently result in different thermal behavior compared with pure particles, as organics would decompose at relatively low temperatures. As shown in Figure 3a, PEI decomposed completely in an N_2_ atmosphere at ~500 °C and the mass kept constant afterwards. Consequently, the adsorption was measured in the decomposing temperature range. Three different pH values, namely 3.2, 6.6, and 10.6, were selected in representative of acid, neutral, and alkaline conditions respectively. Figure 3b–d displays the thermal behavior of the ZSBC-PEI system at different pH values. As the PEI content added was relatively low, the differences of mass loss values among Figure 3b–d were limited. However, the adsorption amount decreased 16.3% and 30.7% in neutral and alkaline solutions respectively, compared with acidic conditions. It clearly indicated that the adsorption content decreased with the increase of pH values, with the highest value corresponding to the acid condition (Table 1), which was in accordance with the zeta potential result (Figure 2). The adsorption of PEI onto the oxide layer was mainly in the form of hydrogen bonding. The higher adsorption amount ensured more effective PEI molecule coverage, thus the surface charge behavior could be effectively altered, and favorable dispersion might be attained in the acid solution.

Since the acidic condition was favored for dispersion, strong acid (hydrogen nitrate) was then used for the powder modification. Shown in Table 1 are the weight losses of acid-etched powders under different pH conditions. The weight losses decreased obviously after treatment. The explanation is as follows: strong acid deteriorated the surface layer by dissolving the oxides (ZrO_2_ and SiO_2_), thus the adsorbed amount of PEI decreased; in such a case dispersion became unstable.

**Table 1:** Weight losses of powders before and after acid-etching treatment.

pH	Weight loss (%)	Weight loss ^a^ (%)
3.2	1.728	1.024
6.6	1.447	0.964
10.6	1.198	0.572

Note: ^a^ after acid etching.

ZrO_2_ + H^+^ → dissolved Zr ions (1)

SiO_2_ + H^+^ → dissolved Si ions (2)

**Figure 4:**
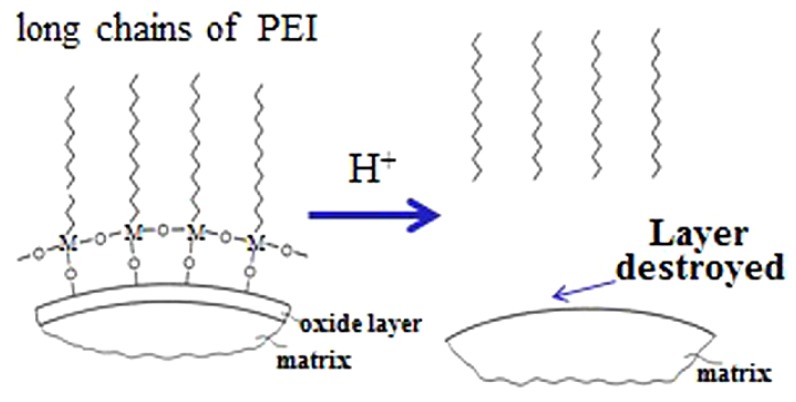
Adsorption and desorption mechanism of PEI on the powder surface.

Figure 4 shows the schematic. Without oxide layers, hydrogen bonding could not be effectively formed, hence favorable dispersion was hampered. It should be noted that although the adsorption amount decreased to a certain extent after etching, the values were still high. This was probably because the surfaces of ZrB_2_, SiC, and B_4_C powders have the tendency to oxidize if exposed to air, resulting in newly-formed surface oxides. Moreover, the highest adsorption amount still appeared in the acidic condition. Hence, it can be summarized that the surface oxide layer, as well as the hydrogen bonding between the layers with PEI, were the decisive factors to obtain a well-dispersed suspension.

### 3.3. Rheological Properties

#### 3.3.1. Rheological Properties of ZSB Powders

**Figure 5:**
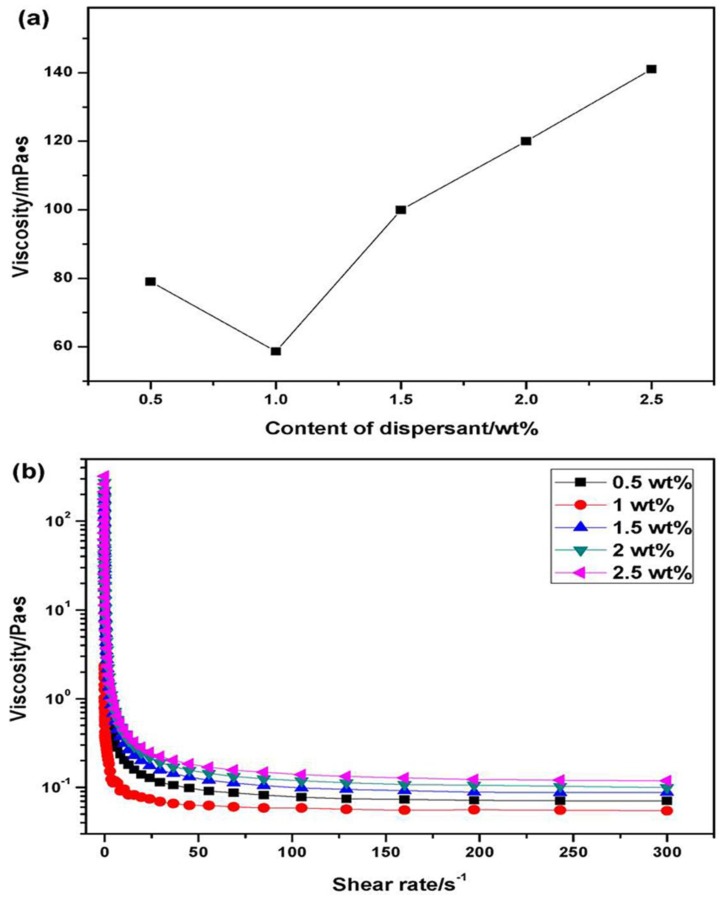
Effect of dispersant content on: (**a**) the viscosity at 100 s^−1^ and (**b**) rheological behavior of 30 vol % ZSB suspensions (pH 3).

For all rheological properties measurements, powders were dispersed in an acidic solution (pH ≈ 3). The viscosity curves of 30 vol % ZSB suspensions are displayed in Figure 5. The viscosity increased from 0.5 wt % to 1 wt %, and decreased thereafter to 2 wt %. The presence of excessive dispersant content was considered to deteriorate the dispersion. An important reason for this was that as the dispersant content increased, the ionic strength of the slurries increased, resulting in the decrease of the surface charge behavior and therefore the increase of the viscosity. The viscosity of ZSB suspension in the presence of 1 wt % PEI was as low as 58.7 mPa·s at 100 s^−1^. Hence, the ZSB suspension could be stabilized with 1 wt % PEI. 

#### 3.3.2. Rheological Properties of ZSBC Powders

**Figure 6:**
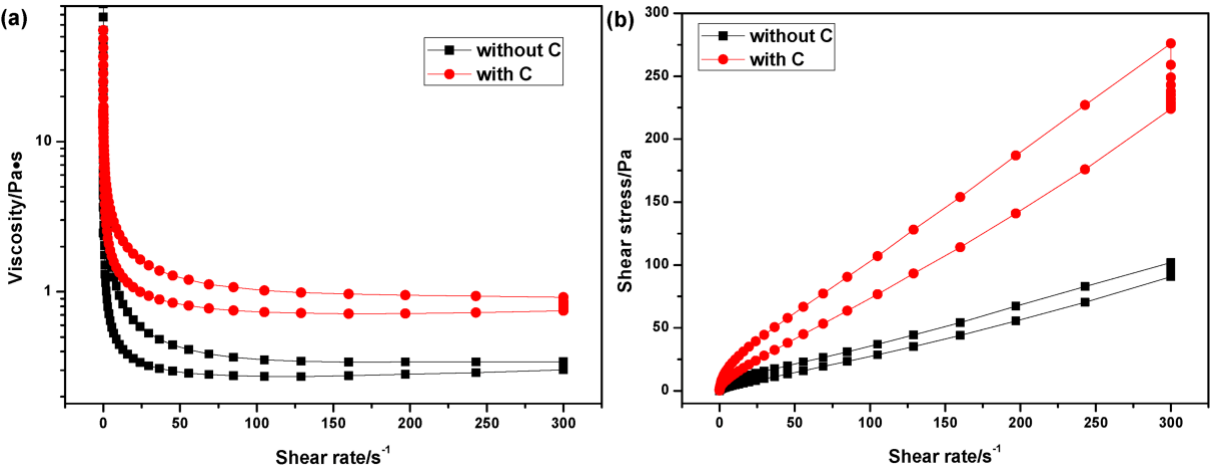
Rheological properties of 40 vol % ZSB and ZrB_2_–SiC–B_4_C–C (ZSBC) suspensions (pH 3).

Since the incorporation of carbon black can effectively remove surface oxide impurity and promote densification progress, additional carbon black was dispersed into water, and the effectiveness of PEI, in dispersing ZSBC powders in aqueous medium, is investigated. The rheological behavior of 40 vol % ZSBC is displayed in Figure 6, and the viscosity curve of 40 vol % ZSB suspensions (without carbon black) is also included for comparison. It can be seen that the dispersion of carbon black significantly increased the viscosity of the slurry (from 0.27 to 0.74 Pa·s, at a shear rate of 100 s^−1^). Meanwhile, the ZSBC suspension showed a more obvious time-dependent behavior compared with the ZSB slurry (Figure 6b), indicating it was less stable than ZSB. This was probably due to the fact that agglomerated structures existed in the ZSBC suspension, as a result of the incorporation of carbon black. Further studies are needed here. Hence, in the current work, the dispersion of carbon black in aqueous media was more difficult than other powders included. For either flow, the viscosity decreased continuously as the shear rate increased (Figure 6a), demonstrating that the slurries were pseudoplastic fluids. Furthermore, both slurries displayed lower viscosities in the descending curve. This was because the particles in the suspensions were re-arranged in an orderly fashion after the constant shearing at 300 s^−1^, thus more homogenized suspension and more stable systems were produced. Generally, flow behavior of ceramic-based flows obey the power-law relationship [19].

(3) $$\tau ={\text{Kr}}^{\text{n}}$$

where *τ* is the shear stress, r is the shear rate, K and n are constants. After fitting of the descending curves, the values of K and n were obtained.

Thus, a slight decrease in n was obtained for ZSB slurry compared with ZSBC, indicating that the former experienced a transition towards more shear thinning behavior. The viscosity of ZSBC slurry at 100 s^−1^ was lower than 1 Pa·s, which was suitable to prepare green bodies via colloidal methods. The shearing thinning can be characterized by the ratio of the viscosities at low and high shear rates,

(4) $$P=\frac{\eta (1s^{-1})}{\eta (10s^{-1})}$$

Then *P* = 1, the suspension shows Newtonian fluid behavior; when *P* > 1, the suspension is a shear thinning flow. The higher value *P* is, the more significant the shear thinning of the suspension. The shear thinning behavior of as-prepared slurries (corresponding to the descending curves) was summarized in Table 2. From the comparison of *P* values, it can be seen that the ZSB suspension exhibited more shear thinning behavior than ZSBC, which coincided well with the above stress-rate fitting results.

**Table 2:** Shear-thinning properties of ZSB and ZSBC slurries.

Composition	*η* (1 s^−1^) (Pa·s)	*η* (10 s^−1^) (Pa·s)	*P*
ZSB	1.535	0.454	3.38
ZSBC	3.880	1.362	2.85

Notes: For ZSB suspension: *K* = 2.54; *n* = 0.43 (standard error < 0.03); For ZSBC suspension: *K* = 4.78; *n* = 0.55 (standard error < 0.02).

## 4. Conclusions

PEI proved to be an effective dispersant for ZSBC powders. Zeta potential measurements demonstrated that suspensions can be stabilized in acidic solution (pH ≈ 3). From TG results, the adsorption decreased with the increase of the pH value. The adsorption mechanism was related to the hydrogen bonding between surface oxide layer and PEI. Strong acid etching treatment played a negative role in the dispersion by destroying the oxide layer. Forty volume percent ZSB(C) suspensions were prepared and exhibited shear-thinning behavior with low viscosity (0.27 Pa·s for ZSB and 0.74 Pa·s for ZSBC, both at 100 s^−1^). The ZSBC suspension showed increased viscosity and more remarkable time-dependent behavior, suggesting the formation of agglomerated structures in the slurry compared with ZSB.
